# Generating Recommendations for Medical Curricula to Reduce Stigma Towards Psychiatry From Medical Students

**DOI:** 10.1192/bjo.2022.110

**Published:** 2022-06-20

**Authors:** Jack Blake, George El-Nimr

**Affiliations:** 1 University Hospital Northwest Midlands, Stoke On Trent, United Kingdom; 2 North Staffordshire Combined Healthcare NHS Trust, Stoke On Trent, United Kingdom; 3 Royal College of Psychiatrists Faculty of Neuropsychiatry, London, United Kingdom; 4 Keele University Medical School, Stoke On Trent, United Kingdom

## Abstract

**Aims:**

In 2021, we completed a project entitled: ‘Stigma Towards Psychiatry: Correlating Personal Experience with Existing Literature’ – ‘International Congress Award Winning’. We aim to use the themes generated from this to inform recommendations to medical educators to reduce stigma in their curricula towards psychiatry.

**Methods:**

Using previously identified themes, we generated a set of recommendations aimed at the pre-admission, pre-clinical and clinical phases of learning. Pre-admission themes include: misconceptions of the role of the psychiatrist and disinterested medical applicants. Pre-clinical themes include: dissociation of psychiatry from medicine and clinical role modelling. Our final category, clinical, includes: cross-speciality support for psychiatry, pathophysiology in de-stigmatisation of psychiatric disease, discrimination of the aspiring psychiatrist, psychiatric exposure in training not seeing conversion of students to psychiatrists and the role of unofficial mentors in continuing enthusiasm for the speciality. Division in this way gave us multiple opportunities to look for areas of potential intervention in influencing medical student's views on psychiatry. Further influencing our recommendations was the feedback from a group of consultant psychiatrists this project was presented to.

**Results:**

Addressing the themes driving stigma from the previous project saw us producing a list of recommendations targeted at each phase of medical school. (1) Pre-admission stage: selecting candidates who are more psychologically minded by recognition of A level psychology as a core subject; encouraging people with experience in mental health settings to apply for medical training (Srivastava et al, 2018); highlighting psychiatry as a medical career in the prospectus. (2) Pre-clinical stage: making students aware of their unconscious stigma towards psychiatry; clearer links made to psychiatry in early medical school to relevant biochemistry, anatomy, physiology and pharmacology (Mahli et al, 2003); tutor / doctor led psychiatry based extracurricular groups; highlighting mental health aspects of functional neurological disorders and disorders within rheumatology such as fibromyalgia. (3) Clinical stage: making clinicians aware of their unconscious stigma towards psychiatry; encouraging cross-speciality support for psychiatry; improving contact with psychiatrists on mental health placements (Archdall et al, 2013).

**Conclusion:**

Stigma towards psychiatry extends from medical school and into clinical practice. It feels important that medical school curricula should be altered in order to change students’ experience of psychiatry within medical school. The recommendations target each stage the themes were identified within. Our local medical school has agreed to let us present this at their curriculum meeting which may pave the way for further refinement and implementation of our suggestions.

